# Supplementary material for: RNAi-mediated rheostat for dynamic control of AAV-delivered transgenes
Source: Nat Commun. 2023 Apr 8;14:1970. doi: 10.1038/s41467-023-37774-5 (PMC10082758; doi:10.1038/s41467-023-37774-5)
Supplement: Supplementary file 1 — Supplementary Information [file 41467_2023_37774_MOESM1_ESM.pdf]

SUPPLEMENTARY TABLE 1: REVERSIR molecules used this study. Legend for chemical modifications is as follows: lower case nucleotides – 2'-*O*-methyl (OMe); upper case nucleotides in parentheses – locked nucleic acid (LNA); (mC) – 5-methyl-cytidine-LNA; dA – 2'deoxyadenosine; L – tri-*N*-acetylgalactosamine.

Compound	Sequence (5 > 3')	Calculated MW	Observed MW
22-mer TTR REVERSIR	a.a.a.a.c.a.g.u.g.u.u.c.u.u.g.(mC.)(T.)(mC.)(T.)a.(T.)adAL	9756.33	9749.763
22-mer NT REVERSIR	a.u.c.a.c.u.a.g.g.u.a.a.u.g.c.(A.)(T.)(A.)(T.)c.(T.)adAL	9751.3	9744.759
9-mer TTR REVERSIR	u.g.(mC)u.(mC)(T)a.(T.)(A)dAL	5131.445	5128.369
9-mer TTR REVERSIR 2	u.g.cu(mC)u(A)u.(A)dAL	5061.267	5058.399
9-mer NT REVERSIR	u.c.(T)a.(T)(A)c.(G.)(T)dAL	5117.417	5114.353
22-mer GLuc REVERSIR	a.u.g.g.g.a.g.u.c.a.a.a.g.u.u.c.(T.)(G.)(T.)u.(T.)gdAL	9842.325	9835.75
9-mer GLuc REVERSIR	u.u.c.(T.)(G.)(T.)u.(T.)gdAL	5161.573	5158.252

SUPPLEMENTARY TABLE 2: GalNAc-siRNA conjugates used in this study. Legend for chemical modifications is as follows: lower case nucleotides - 2'-*O*-methyl (OMe); upper case nucleotides - 2'-deoxy-2'-fluoro (F); . – phosphorothioate linkage; L – tri-*N*-acetylgalactosamine.

Compound	Strand	Sequence (5' > 3')	Calculated MW	Observed MW
TTR siRNA	AS	u.U.auaGagcaagaAcAcuguu.u.u	7656.116	7652.169
	S	a.a.caguGuUCUugcucuauaaL	8686.456	8681.992
GLuc siRNA	AS	a.C.aaaCaGAacuuUgAcuccc.a.u	7573.067	7569.176
	S	g.g.gaguCaAAGuucuguuuguL	8798.502	8793.994
Transgene regulator siRNA 1	AS	a.C.gcgAuAAauagUcGuacua.g.u	7670.096	7666.167
	S	u.a.guacGaCUAuuuaucgcguL	8702.454	8697.987
Transgene regulator siRNA 2	AS	a.C.gcgAuAAaaauAuCugggu.c.g	7709.135	7705.19
	S	a.c.ccagAuAUUuuuaucgcguL	8662.428	8657.981
Transgene regulator siRNA 3	AS	a.C.gauAcGUaaagAcGacuuu.u.u	7631.059	7627.145
	S	a.a.agucGuCUUuacguaucguL	8702.453	8697.987
TMPRSS6 siRNA control	AS	u.U.gGaGgCcAcagUcAcAgUg.c.u	7640.979	7637.102
	S	c.a.CuGuGaCUGuGgCcUcCaAL	8631.246	8626.89

SUPPLEMENTARY TABLE 3: Differential gene expression analysis of *in vitro* RNAseq data

Hep3B	Number of differentially-expressed genes		Strongest DEG (% KD)	Number of 2-fold off-targets
	Up-regulated	Down-regulated		
**Transgene regulator siRNA 1**	0	0	NA	0
**Transgene regulator siRNA 2**	0	1	-30%	0
**Transgene regulator siRNA 3**	0	0	NA	0

Primary mouse hepatocytes	Number of differentially-expressed genes		Strongest DEG (% KD)	Number of 2-fold off-targets
	Up-regulated	Down-regulated		
**Transgene regulator siRNA 1**	13	9	178%	0
**Transgene regulator siRNA 2**	0	1	-41%	0
**Transgene regulator siRNA 3**	44	66	-66%	5

Supplementary Figure 1

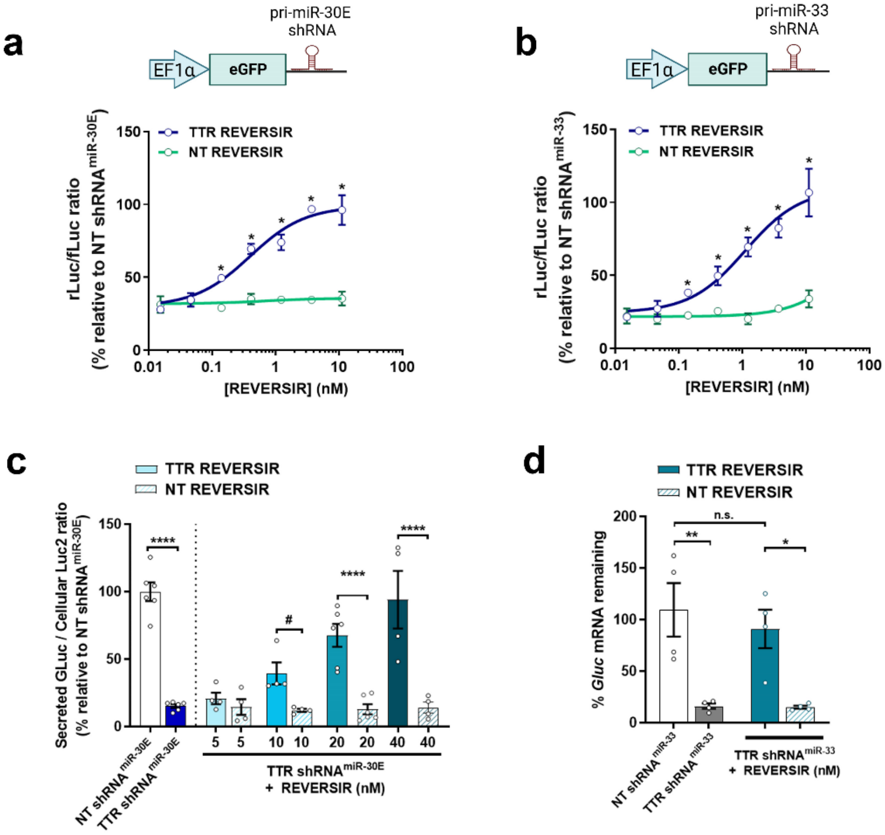

SUPPLEMENTARY FIGURE 1: Additional *in vitro* and *in vivo* analyses supporting AAV regulatory switch leveraging intronically-expressed shRNA and REVERSIR

(a and b) *In vitro* assessment of REVERSIR-mediated reversal of target silencing by miRNA-scaffolded shRNA in the dual luciferase reporter assay. Cos7 cells were co-transfected for 48 hours with luciferase reporter plasmid and GFP marker constructs expressing **(a)** miR-30E- or **(b)** miR-33-embedded TTR or NT shRNAs, along with increasing concentrations of 22-mer TTR or matched NT REVERSIR (n=4 replicates per dose from two independent experiments). Statistical analysis was performed using multiple unpaired t-test by row and correction for multiple comparisons using Holm-Sidak method (*$p<0.05$). **(c)** Validation of GLuc transgene self-silencing with intronic miR-30E-shRNA-containing AAV constructs and subsequent induction with increasing doses of REVERSIR in HepG2 cells. AAV constructs were co-transfected with a Luc2 control plasmid for normalization at 5:1 molar ratio. GLuc and FLuc intensities were assayed in cell culture supernatant and lysate, respectively, and GLuc/FLuc ratios expressed as % relative to NT shRNA-expressing AAV plasmid. Statistical analysis was performed using ordinary one-way ANOVA [$F(9, 38) = 21.09$; $p<0.0001$] followed by Bonferroni's post hoc test for multiple comparisons (****$p<0.0001$). 10nM REVERSIR conditions were additionally compared using unpaired two-tailed *t*-test (#, p=0.016). **(d)** Quantification of *Gluc* mRNA levels by qRT-PCR in HepG2 cells 48h following transfection with indicated AAV plasmids and REVERSIR. GLuc transcript levels were normalized to *Luc2* mRNA as internal transfection control. Data were analyzed using ordinary one-way ANOVA [$F(3, 12) = 9.54$; p=0.0017) followed by Tukey's multiple comparisons test (*$p<0.05$ **$p<0.01$; n=4 replicates from two experiments). All error bars represent s.e.m. Schematics of shRNA expression constructs shown in **(a)** and **(b)** were made using BioRender.com. Raw data from individual experimental replicates along with detailed statistical analyses are provided as a Source data file.

Supplementary Figure 2

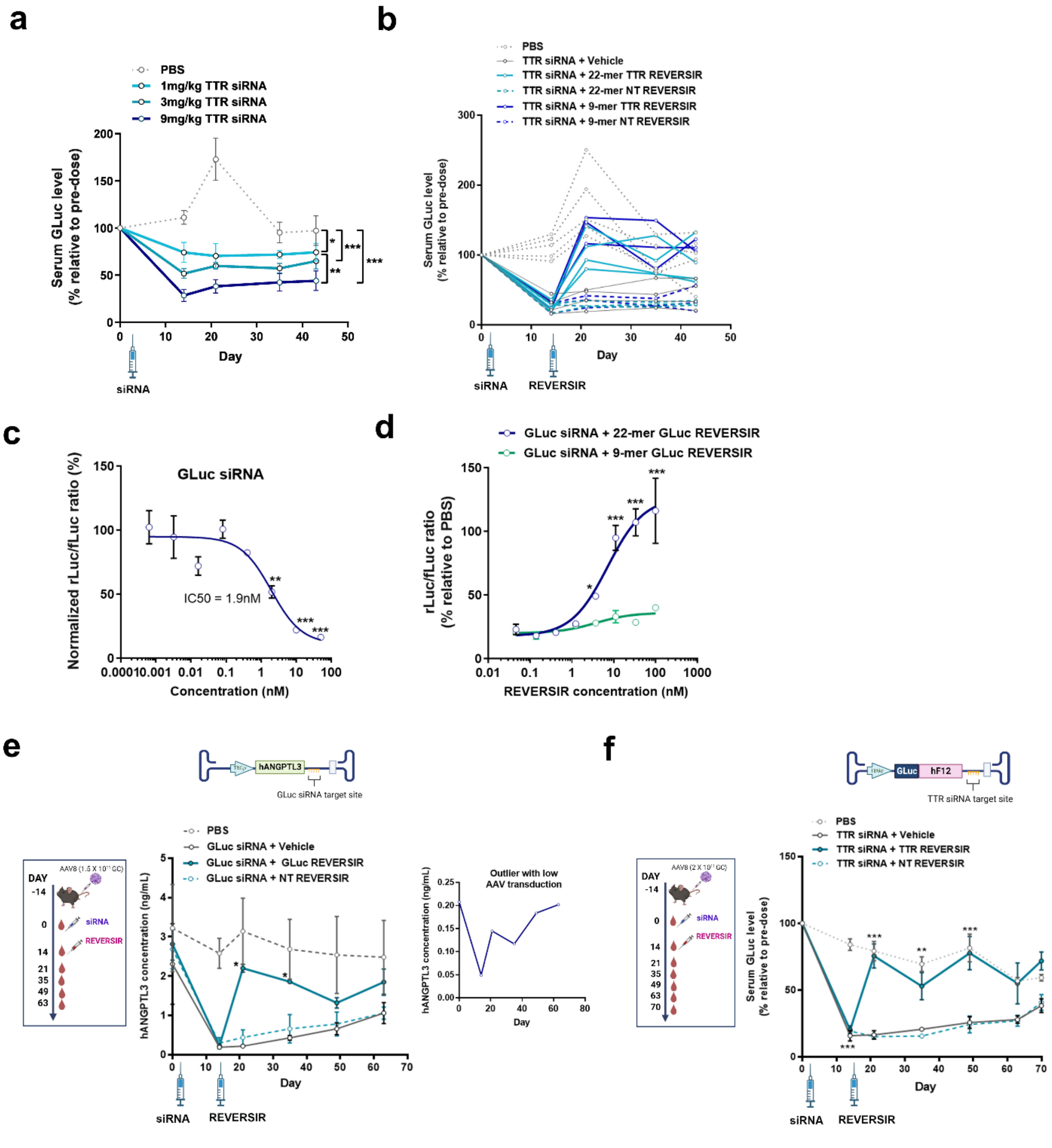

SUPPLEMENTARY FIGURE 2: Additional *in vitro* and *in vivo* analyses supporting AAV regulatory switch leveraging exogenous siRNA and REVERSIR

(a – c) Panels represent data from individual animals or additional groups tested as part of the study shown in Figures 3b and 3c. PBS and 9mg/kg siRNA conditions are identical to those shown in Figure 3b. **(a)** Dose-dependent knockdown of serum GLuc levels in AAV-injected mice treated with 1 (*n*=3), 3 (*n*=4), and 9mg/kg (*n*=4) TTR siRNA as compared to PBS control (*n*=5). Data were analyzed by ordinary one-way ANOVA [F(3,12) = 26.62; $p<0.0001$] with Tukey's post hoc test (D14 *$p<0.05$ **$p<0.01$ ***$p<0.001$). **(b)** Averaged data in Figure 3c presented as spaghetti plot. **(c)** On-target silencing activity of GLuc siRNA in dual luciferase reporter system (*n*=4 replicates per dose). Statistical analysis was performed using one-way ANOVA [F(8,27) = 13.38; $p<0.0001$] followed by Dunnett's multiple comparisons test relative to PBS control (**$p<0.01$ ***$p<0.001$). **(d)** Normalized on-target luciferase activity 48h following co-transfection of Cos7 cells with 10nM GLuc siRNA and increasing doses of 22-mer or 9-mer GLuc REVERSIR. Data were analyzed by two-way ANOVA with Dunnett's correction (*$p<0.05$ ***$p<0.001$). Effect of REVERSIR dose [F(8,22) = 28.3; p<0.0001] and REVERSIR length [F(1,22) = 79.6; $p<0.0001$]. **(e)** Regulation of hANGPTL3 transgene**.** AAV-injected mice were treated with 9mg/kg GLuc siRNA (D0), after which 1.6mg/kg 9-mer GLuc REVERSIR or NT REVERSIR was administered on D14. Plasma hANGPTL3 concentrations were assessed by ELISA (*n*=3 mice for PBS, vehicle, and NT REVERSIR groups; *n*=2 mice for GLuc REVERSIR group). **(f)** Regulation of GLuc-hF12 transgene. AAV-transduced mice were treated with 9mg/kg TTR siRNA on D0, then given 1.6mg/kg TTR REVERSIR or NT REVERSIR (D14). Serum GLuc was measured and plotted relative to pre-treatment with siRNA (*n*=3 mice per group). Data in (e) and (f) were analyzed using two-way repeated measures ANOVA followed by Dunnett's and Tukey's post hoc tests, respectively. *$p<0.05$ **$p<0.01$ ***$p<0.001$ n.s., not significant. Error bars represent s.e.m. Source data are provided as a Source Data file. Diagrams were created with BioRender.com.

Supplementary Figure 3

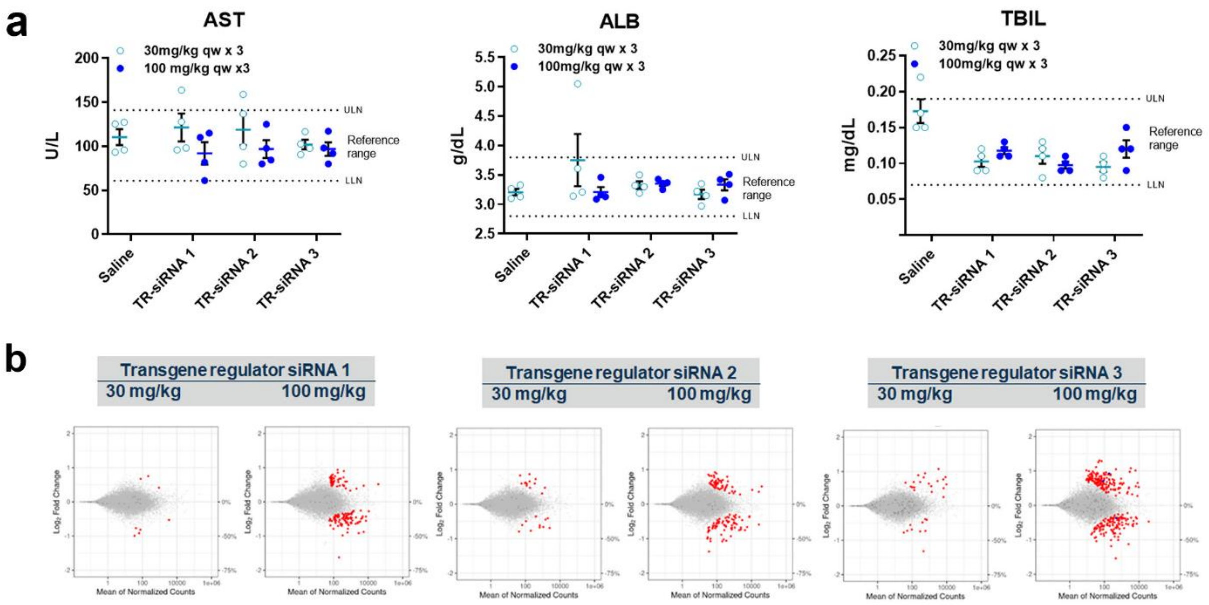

SUPPLEMENTARY FIGURE 3: Minimal liver function test (LFT) elevations and transcriptional dysregulation in rat toxicity studies of transgene regulator siRNAs

(a) Serum levels of aspartate aminotransferase (AST), albumin (ALB), and total bilirubin (TBIL) at necropsy (D16). Data were analyzed using ordinary one-way ANOVA with Dunnett's correction. AST [$F(6,21) = 0.93$; $p=0.50$], ALB [$F(6,21) = 1.19$; $p=0.35$], TBIL [$F(6,21) = 7.19$; $p=0.0003$; all groups significantly lower than saline control] ($n=4$ males per group). Error bars represent s.e.m. **(b**) RNA-seq assessment of rat livers collected 24 h after last dose. Log_2 fold change plots (MA plot) represent the average signal from each cohort ($n=4$ males). Dots represent individual rat transcripts, their average number of counts and relative change in expression compared to control group dosed with 0.9% NaCl. Gray dots represent gene transcripts that were not found to be differentially expressed following siRNA treatment compared to control. Blue and red dots represent differentially expressed transcripts (false discovery rate < 0.05) with or without a canonical match (8mer, 7mer-m8, 7mer-A1) to the antisense seed region, respectively. Dots in dark gray represent transcripts harboring a canonical seed-matched site but that were not found to be differentially expressed. Source data are provided as a Source data file.
