# Supplementary material for: RNAi-mediated rheostat for dynamic control of AAV-delivered transgenes
Source: Nat Commun. 2023 Apr 8;14:1970. doi: 10.1038/s41467-023-37774-5 (PMC10082758; doi:10.1038/s41467-023-37774-5)
Supplement: Supplementary file 3 — Description of Additional Supplementary Files [file 41467_2023_37774_MOESM3_ESM.pdf]

Description of Additional Supplementary Files

File Name: Supplementary Data 1
Description: RNAi target site, shRNA, and dual luciferase reporter insert sequences
