# Supplementary material for: RNAi-mediated rheostat for dynamic control of AAV-delivered transgenes
Source: Nat Commun. 2023 Apr 8;14:1970. doi: 10.1038/s41467-023-37774-5 (PMC10082758; doi:10.1038/s41467-023-37774-5)
Supplement: Supplementary file 5 — Reporting Summary [file 41467_2023_37774_MOESM5_ESM.pdf]

nature portfolio

Corresponding author(s): Vasant Jadhav

Last updated by author(s): Mar 8, 2023

Reporting Summary

Nature Portfolio wishes to improve the reproducibility of the work that we publish. This form provides structure for consistency and transparency in reporting. For further information on Nature Portfolio policies, see our Editorial Policies and the Editorial Policy Checklist.

Statistics

For all statistical analyses, confirm that the following items are present in the figure legend, table legend, main text, or Methods section.

n/a	Confirmed	
☐	☒	The exact sample size (*n*) for each experimental group/condition, given as a discrete number and unit of measurement
☐	☒	A statement on whether measurements were taken from distinct samples or whether the same sample was measured repeatedly
☐	☒	The statistical test(s) used AND whether they are one- or two-sided<br>*Only common tests should be described solely by name; describe more complex techniques in the Methods section.*
☒	☐	A description of all covariates tested
☐	☒	A description of any assumptions or corrections, such as tests of normality and adjustment for multiple comparisons
☐	☒	A full description of the statistical parameters including central tendency (e.g. means) or other basic estimates (e.g. regression coefficient) AND variation (e.g. standard deviation) or associated estimates of uncertainty (e.g. confidence intervals)
☐	☒	For null hypothesis testing, the test statistic (e.g. *F*, *t*, *r*) with confidence intervals, effect sizes, degrees of freedom and *P* value noted<br>*Give P values as exact values whenever suitable.*
☒	☐	For Bayesian analysis, information on the choice of priors and Markov chain Monte Carlo settings
☒	☐	For hierarchical and complex designs, identification of the appropriate level for tests and full reporting of outcomes
☒	☐	Estimates of effect sizes (e.g. Cohen's *d*, Pearson's *r*), indicating how they were calculated

Our web collection on statistics for biologists contains articles on many of the points above.

Software and code

Policy information about availability of computer code

Data collection	Dual Glo and Gaussia luciferase assays were read using SpectraMax m5e and SpectraMax L plate readers, respectively.
Data analysis	Luciferase assays were analyzed using SoftMax Pro 7.1 software.<br>Figure preparation and statistical analyses were conducted using GraphPad Prism v.7.<br>RNA-seq reads were filtered using ea-utils software fastq-mcf v1.05 (https://expressionanalysis.github.io/ea-utils/). Filtered reads were aligned to the genome using STAR (ultrafast universal RNAseq aligner) v2.7.9a. Uniquely aligned reads were counted by featureCounts v2.0.2.<br>Differential expression analysis was conducted using R package DESeq2 v1.34.0 on R version 4.1.0.<br>Decamers for identification of transgene regulator siRNA sequences were aligned to species transcriptomes using BLASTN.

For manuscripts utilizing custom algorithms or software that are central to the research but not yet described in published literature, software must be made available to editors and reviewers. We strongly encourage code deposition in a community repository (e.g. GitHub). See the Nature Portfolio guidelines for submitting code & software for further information.

Data

Policy information about availability of data

All manuscripts must include a data availability statement. This statement should provide the following information, where applicable:

- Accession codes, unique identifiers, or web links for publicly available datasets
- A description of any restrictions on data availability
- For clinical datasets or third party data, please ensure that the statement adheres to our policy

The raw RNAseq data presented in Figure 4b and Supplementary Figure 3b have been deposited to the NCBI Gene Expression Omnibus and are accessible through GEO Series accession number GSE214065 (https://www.ncbi.nlm.nih.gov/geo/query/acc.cgi?acc=GSE214065). MicroRNA seed sequences were obtained from the miRbase database.

Human research participants

Policy information about studies involving human research participants and Sex and Gender in Research.

Reporting on sex and gender	N/A
Population characteristics	N/A
Recruitment	N/A
Ethics oversight	N/A

Note that full information on the approval of the study protocol must also be provided in the manuscript.

Field-specific reporting

Please select the one below that is the best fit for your research. If you are not sure, read the appropriate sections before making your selection.

☒ Life sciences ☐ Behavioural & social sciences ☐ Ecological, evolutionary & environmental sciences

For a reference copy of the document with all sections, see nature.com/documents/nr-reporting-summary-flat.pdf

Life sciences study design

All studies must disclose on these points even when the disclosure is negative.

Sample size	No statistical sample size calculation was performed. Sample sizes for each experiment are detailed either in the relevant figure legends or source data file. In vitro studies utilized N>=3 biological replicates and in vivo studies generally utilized 3-5 animals per group per 3R's principles. N>=3 is sufficient to conduct significance testing by t-test, one-way ANOVA, or two-way ANOVA.
Data exclusions	In Figure 4a, one replicate in the TR-siRNA 2 - off-target reporter (1 seed-matched site) group was removed as an outlier due to being significantly different from other observations. Luciferase readings suggest that the well was misdosed and did not receive siRNA at transfection. Replicate was also identified as an outlier based on Grubbs test (please see Source data file; Figure 4a for detailed information). In Figure 3f, one animal showed basal hANGPTL3 transduction that was significantly low, with levels comparable to that typically observed at 10-fold lower viral titer. This suggests a likely issue with tail vein dosing. This animal is omitted from the main Figure 2f and shown separately in Supplementary Figure 2e. Data from this animal was included for statistical analyses, with detailed information provided in the Source data. No other data were excluded from other datasets.
Replication	Cell culture studies in 1d, 4a and Supplementary Figures 1a, 1b, 1c, 1d were repeated two or more times, with N>=2 wells per experiment. All attempts at replication were successful. Experiment shown in Figure 1c was performed once with N=6 wells per group with each well monitored over time. Cell culture studies presented in Supplementary Figures 2c and 2d were performed once with N>=2 wells per condition using dual-reporter plasmids to enhance consistency and reproducibility. Data from individual experiments are reported in the Source data for the relevant figures.<br><br>The described animal studies were not replicated per 3Rs principles. Data from individual animals for all in vivo studies have been reported in the Source data, along with statistical analyses. In vivo studies shown in Figures 1e, 1g, 2c, 2d, and Supplementary Figure 2f utilized the same test article reagents (TTR siRNA, TTR REVERSIR) across multiple AAV transgenes, with the overall finding of transgene KD and REVERSIR-mediated recovery of expression reproduced across the studies. Supplementary Figure 2e utilized a different siRNA/REVERSIR pair targeting GLuc, demonstrating that the approach is not exclusive to use of TTR siRNA/shRNA and TTR REVERSIR.
Randomization	For in vivo studies, animals were randomized based on body weight and randomly assigned to control or treatment groups. For in vitro studies, wells were plated with equal numbers of cells and randomly assigned to different treatment groups.
Blinding	No blinding was performed in this study. In most cases, rodent dosing and blood/tissue collections were performed by one group and samples

Blinding	transferred to another group for analysis. Most measurements in this study were made using unbiased assays, such as luciferase assays from cell culture lysate or supernatant, or luciferase or ELISA assays from mouse serum/plasma.

Reporting for specific materials, systems and methods

We require information from authors about some types of materials, experimental systems and methods used in many studies. Here, indicate whether each material, system or method listed is relevant to your study. If you are not sure if a list item applies to your research, read the appropriate section before selecting a response.

Materials & experimental systems

n/a	Involved in the study
☐	☒ Antibodies
☐	☒ Eukaryotic cell lines
☒	☐ Palaeontology and archaeology
☐	☒ Animals and other organisms
☒	☐ Clinical data
☒	☐ Dual use research of concern

Methods

n/a	Involved in the study
☒	☐ ChIP-seq
☒	☐ Flow cytometry
☒	☐ MRI-based neuroimaging

Antibodies

Antibodies used	Human ANGPTL3 ELISA kit: R&D Systems Catalog #DANL30 Lot #244991 - Assay utilizes a polyclonal antibody specific for human ANGPTL3 conjugated to horseradish peroxidase.<br>Mouse EPO Quantikine ELISA kit: R&D Systems Catalog #MEP00B - Assay utilizes a monoclonal antibody specific for mouse Epo conjugated to horseradish peroxidase.
Validation	All ELISA kits are commercially available and have been previously validated by the manufacturer. The human ANGPTL3 ELISA kit listed above recognizes natural and recombinant human ANGPTL3. No significant cross-reactivity or interference was observed with recombinant human angiopoietin-1/2/4, human angiopoietin-like 1/2/4/7, mouse and mouse angiopoietin-like 3/4/7. The mouse EPO ELISA kit listed above recognizes natural and recombinant mouse EPO. No significant cross-reactivity or interference was observed to several other serum components.

Eukaryotic cell lines

Policy information about cell lines and Sex and Gender in Research

Cell line source(s)	Cos-7: ATCC CRL-1651<br>HepG2: ATCC HB-8065<br>Hep3B: ATCC HB-8064<br>Primary mouse hepatocytes: BIOIVT, Cat # M005052-P, Lot GBW (Male ICR/CD-1 Mouse Hepatocytes – Pooled)
Authentication	Cos-7, HepG2, and Hep3B cells used in this study were directly obtained from ATCC, where they have been authenticated using STR profiling. Cryoplateable mouse hepatocytes were determined by the provider to be >80% viable with a minimum of 5 million cells per vial by Trypan Blue exclusion.
Mycoplasma contamination	Cos-7, HepG2, and Hep3B cells were certified to be mycoplasma-free on purchase from ATCC. However, the cell lines were not tested for mycoplasma contamination by the investigators during the course of the study. All cell lines were maintained for 10-12 passages, after which they were discarded and fresh low-passage stocks were thawed.
Commonly misidentified lines<br>(See ICLAC register)	No misidentified cell lines were used.

Animals and other research organisms

Policy information about studies involving animals; ARRIVE guidelines recommended for reporting animal research, and Sex and Gender in Research

Laboratory animals	Mouse (C57BL/6, female, 6 - 8 weeks), Rat (Sprague Dawley, male, 6 - 8 weeks); Obtained from Charles River Laboratories. Animals were housed in a temperature-controlled environment maintained at 20 – 26°C and 30 - 70% humidity under standard 12:12h light/dark cycles.
Wild animals	This study did not involve wild animals.
Reporting on sex	Female mice were used for all in vivo AAV transduction studies for consistency and for ease of group housing. Male rats were used for in vivo hepatotoxicity evaluations.
Field-collected samples	This study did not utilize field-collected samples.

Ethics oversight	All rodent studies were conducted using protocols consistent with local, state and federal regulations, as applicable, and approved by the Institutional Animal Care and Use Committee (IACUC) at Alnylam Pharmaceuticals. We have also provided an ethics statement describing this information in the METHODS section of the manuscript.

Note that full information on the approval of the study protocol must also be provided in the manuscript.
